# PTH2R is related to cell proliferation and migration in ovarian cancer: a multi-omics analysis of bioinformatics and experiments

**DOI:** 10.1186/s12935-022-02566-2

**Published:** 2022-04-11

**Authors:** Wang Xiaowei, Lu Tong, Qu Yanjun, Fan Lili

**Affiliations:** 1 Department of Ultrasnography in Gynecology and Obstetrics, The First Affiliated Hospital of Harbin Medical University, Harbin, China; 2 Department of Children’s and Adolescent Health, Public Health College, Harbin Medical University, Harbin, China; 3 Department of Thoracic Surgery, The Second Affiliated Hospital of Harbin Medical University, Harbin, China

**Keywords:** Ovarian cancer, PTH2R, TCGA, GEO, Multi-omics

## Abstract

**Background:**

Ovarian cancer is a common gynecological disease and seriously endangers women's health. Currently, there is still a lack of effective molecular markers for the diagnosis and treatment of ovarian cancer. The present study aimed to investigate the molecular markers associated with ovarian cancer.

**Methods:**

The molecular and gene related to ovarian cancer were extracted from GEO database and TCGA database by bioinformatics, and the related genes and functions were further analyzed. The results were verified by qPCR, WB, CCK-8 and Transwell experiments.

**Results:**

Data analysis showed that PTH2R gene was highly expressed in tumors, and 51 HUB genes were obtained. Finally, experimental verification showed that PTH2R gene was highly expressed in ovarian cancer, and PTH2R gene was involved in the proliferation, invasion and metastasis of ovarian cancer cells.

**Conclusions:**

After experimental verification, we found that knocking down the expression of PTH2R can inhibit the proliferation, invasion and migration of tumor cells.PTH2R is expected to become a new molecular marker for ovarian cancer.

**Supplementary Information:**

The online version contains supplementary material available at 10.1186/s12935-022-02566-2.

## Background

Ovarian cancer is one of the three malignant gynecological tumors. Although ovarian cancer is less common than either cervical or endometrial cancers, its mortality rate exceeds that of cervical and endometrial cancers combined. [1, 2] The 5-year survival rate for ovarian cancer is < 40%, making it one of the deadliest gynecological tumors. [3] Its lethality mainly arises from its aggressive nature and from the difficulty of achieving early diagnosis. As a result, most patients develop highly metastatic, invasive disease in later stages [4]. Ovarian cancer is highly heterogeneous and adenocarcinoma accounts for the majority of malignant tumors. Current treatment strategies include platinum- and taxane-based chemotherapy, as well as neoadjuvant chemotherapy after surgical resection. Unfortunately, most patients relapse or develop drug resistance within 36 months. [5] At present, the molecular etiology of the ovarian cancer remains elusive; thus, finding effective biomarkers for the diagnosis and treatment of ovarian cancer is a priority.

In recent years, with the development of sequencing technology, bioinformatics has come to play an important role in revealing the occurrence and development of tumors [6, 7]. In the past, many researchers did not have direct access to sequencing data, due to sample or funding constraints. Now, however, an increasing number of researchers are uploading their microarray or sequencing data to public databases, allowing oncology-related researchers around the world to download and process data to reveal the underlying pathogenesis of tumors. Among these, the Gene Expression Omnibus (GEO) [8] and The Cancer Genome Atlas (TCGA) databases [9] are the most widely used. The GEO database contains raw microarray and sequencing data uploaded by numerous researchers, as well as data from a variety of molecular types, including mutation, messenger ribonucleic acid (mRNA), non-coding RNA, and other transcriptome and methylation data. A large number of studies have analyzed public data from GEO; many have used multi-data set joint analysis to find important regulatory molecules. For instance, Bi et al. used GSE17260 and GSE73614 to develop a glycolysis-related prognostic signature in ovarian cancer [10]. In addition, Jin et al. identified CXCL10 gene as promising biomarker for ovarian cancer immunotherapy [11]. TGCA database is also widely used in oncology studies; it is a pan-cancer project hosted by the National Institutes of Health (NIH), providing a wide variety of tumors and different molecular data types that can be downloaded and analyzed. In the past few years, most studies have focused on RNA sequencing (RNA-Seq) analysis, such as mRNA and non-coding RNAs [12–15]. Recently, following the updating of old algorithms and the discovery of new ones, CIBERSORT, MCPcounter, and other algorithms have been used to assess tumor immune cell infiltration. Meanwhile, ESTIMATE has been used to assess the scores of Immune and stromal cells in the tumor microenvironment [16–18].

Exploring public databases has revealed many molecules that are highly expressed in ovarian cancer, such as CXCL10 [11], MMP16 [19], MCUR1 [20], MRPL15 [21] etc. In combination with multiple data sets, the expression of the gene Parathyroid Hormone 2 Receptor (PTH2R) has been shown to be significantly elevated in ovarian cancer. To further mine PTH2R-related data, here relevant information was classified into high or low expression PTH2R groups, according to the expression value of PTH2R. Further analyses of these groups, such as mutation characteristics, copy number variation (CNV), drug resistance characteristics, and immune infiltration, were conducted to explore PTH2R-related genes and functions from the perspective of various data. Furthermore, quantitative polymerase chain reaction (qPCR), western blot (WB), Cell Counting Kit 8 (CCK-8), and transwell assays were conducted alongside other experiments to verify the expression and function of PTH2R. Bioinformatics analysis and experimental verification revealed that the high expression of PTH2R can promote the growth, invasion, and metastasis of ovarian cancer. PTH2R may therefore be useful as a potential biomarker for ovarian cancer in the future.

## Materials and methods

### Sample source

Ovarian cancer tissue (n = 12) and normal ovarian tissue (n = 12) samples were collected from gynecology department in The First affiliated Hospital of Harbin Medical University, from 3 to 9 months in 2020. None of the patients were treated before undergoing surgery. The surgically resected specimens were immediately placed in liquid nitrogen and then transferred to a −80 °C refrigerator for storage. All patients in the study provided written informed consent for the biological study. The research protocol (including specimen collection) was reviewed and approved by the Biomedical Ethics Committee of The First Affiliated Hospital of Harbin Medical University (Batch Number: 2022JS01), All procedures were conducted in accordance with the Guidelines of the World Medical Association Declaration of Helsinki. The clinicopathological staging and typing of the patients met the Joint Council on Cancer (AJCC) typing criteria.

### Public data acquisition and preprocessing

Using R software (Version 4.1.0, http://r-project.org/) the “GEOquery” package [22] from the GEO database (https://www.ncbi.nlm.nih.gov/geo/) was applied to download the GSE18520 and GSE66957 ovarian expression datasets. The samples in these datasets were sourced from *Homo sapiens*, and the platform is based on the GPL570 (HG-U133_Plus_2) Affymetrix Human Genome U133 Plus 2.0 Array. The GSE18520 dataset includes 63 samples from 53 ovarian cancer patients and 10 normal samples. GSE66957 includes 57 samples and 12 Normal-ovarian samples from 69 ovarian cancer patients within the dataset. All these data were included in this study.

In addition, count data of ovarian cancer RNA-Seq, single nucleotide polymorphism (SNP) data, and matching clinical data (n = 379) were downloaded from TCGA database using Genomic Data Commons (GDC) software (https://portal.gdc.cancer.gov/projects/). As there is no normal control for ovarian cancer in TCGA, here the obtained TCGA data were combined with GTEx to obtain normal ovarian control download samples (n = 88) and ovarian cancer samples (n = 427). RNA-Seq count data were obtained through the University of California Santa Cruz (UCSC) Xena browser (https://xenabrowser.net/datapages/; the data were corrected in batches).

### Screening of differentially expressed genes (DEGs)

The differentially expressed genes (DEGs) of the GSE118520 dataset were downloaded through the R package “limma” [23], following which the package “ggplot2” was used to draw a volcano map of the DEGs to show their differential expressions. DEGs were considered significant when they met the thresholds of *P* < 0.05 and |log2FoldChange|> 1. Subsequently, DEGs in ovarian cancer and normal samples in the combined TCGA-GTEx dataset were screened using the R package “Deseq2” [24], using the same thresholds as detailed above. Taking the intersections of the DEGs obtained from the two data sets, the candidate gene of interest was then selected for subsequent analysis.

### Mutation and CNV analysis

The somatic mutation data of TCGA-OV patients were extracted by using the R package “maftools” [25]. Somatic mutation data of patients in the high and low gene expression groups were then collected and analyzed.

To analyze the changes in CNVs in TCGA-OV patients within the high gene expression group, the R package “TCGAbiolinks” [26] was used to download the “Masked Copy Number Segment” data of patients. GISTIC 2.0 analysis of the downloaded CNV fragments was then conducted through GenePattern (https://cloud.genepattern.org) [27].

### Weighted gene co-expression network analysis (WGCNA)

The R package “WGCNA” [28] was used to analyze the GSE18520 and TCGA-OV datasets. The samples were divided into high and PTH2R low expression groups. The standardized data were then used to construct a co-expression network. For all functions in WGCNA, the correlations of double weights were used as the correlation method. A topological overlap metric (TOM) was used for network construction and module identification. The calculation parameters minModuleSize = 50 and mergeCutHeight = 1,000 were used to analyze data. Ultimately, the hub genes were obtained from the intersection of the genes in the module with the highest significance, and using the previously obtained DEGs.

### Functional enrichment analysis

Gene ontology (GO) analysis is commonly used to conduct large-scale functional enrichment studies, including biological process (BP), molecular function (MF), and cellular component (CC) [29]. The Kyoto Encyclopedia of Genes and Genomes (KEGG) is a widely used database that stores information about genomes, biological pathways, diseases, and drugs [30]. Here, GO annotation and KEGG pathway enrichment analyses were performed on the hub gene using the R package “clusterProfiler” [31]. A critical value of false discovery rate (FDR) < 0.05 was considered to imply statistical significance.

Gene Set Enrichment Analysis (GSEA) is a calculation that analyzes whether a particular set of genes is statistically different between two biological states. It is commonly used to estimate changes in the activities of pathways and biological processes in sample expression datasets. Here, GSEA was conducted to study the differences in biological processes between groups based on the gene expression profile data set of TCGA-OV patients [32]. The gene set “c2.cp.kegg.v7.2.symbols” was downloaded from the MSigDB database [33] for GSEA, and FDR < 0.25 and *P* < 0.05 were considered to represent a significant enrichment.

### Drug sensitivity analysis

The CellMiner database (https://discover.nci.nih.gov/cellminer/) is a web-based tool that contains genomic and pharmacological information for researchers to use transcripts and drug response data from the NCI-60 cell line [34]. The data were compiled by the National Cancer Institute. CellMiner provides transcriptional expression levels for the drug responses of 22,379 genes, 360 microRNAs, and 20,503 compounds [35]. The mRNA expression profiles and drug activity data including the PTH2R gene were downloaded from the CellMiner database. The correlation between PTH2R gene expression and compound sensitivity was calculated through Pearson’s correlation analysis. *P* < 0.05 was considered to represent statistical significance.

The Genomics of Drug Sensitivity in Cancer (GDSC) database(www.cancerrxgene.org/)can be used to search for tumor drug response data and genome sensitive markers [36]. Here, the pRRophetic algorithm [37], the ridge regression model, and IC50 were used to predict the sensitivities of the high and low PTH2R expression groups to common anticancer drugs.

### Immune cell infiltration analysis and tumor immunoanalysis

CIBERSORT (http://CIBERSORT.stanford.edu/) and the LM22 characteristic gene matrix were used to predict the proportions of 22 immune cells in all samples within the predicted dataset [38]. CIBERSORT was used to assess the abundances of 22 immune cells in TCGA-OV dataset, and to calculate the correlations between these 22 kinds of immune cells. Then, by integrating candidate gene expression, spearman’s correlations were calculated between the gene expression and these immune-infiltrating cells, with *P* < 0.05 being considered to represent statistical significance.

### Cell culture

The following three cell lines were used in this study: the human ovarian cancer cell lines SK-OV3 and A2780, normal ovarian surface epithelium cell line IOSE-80. All cell lines were purchased from the American Type Culture Collection (Manassas, VA, USA). All cells were cultured in high glucose Dulbecco’s modified Eagle’s Medium (DMEM, Corning) treated with 10% fetal bovine serum (FBS; Hyclone) and 1% penicillin/streptomycin solution (Invitrogen) in a 37 °C humidity, 5% CO_2_ incubator.

### Real-time fluorescence qPCR

Total RNA was extracted using RNAiso Plus reagent (Takara Bio, Kusatsu, Japan). RNA concentration and purity were assessed using a NanoDrop 2000 system (Thermo Fisher, Carlsbad, CA, USA). Reverse transcription was then performed using the PrimeScript™ RT reagent kit with gDNA eraser (Perfect Real Time; Takara Bio). The SYBR^®^ Premix Ex AQ ™ II (Tli RNaseH Plus; Takara Bio) in ABI 7500 Fast System (Life Technologies, Carlsbad, CA, USA) was used for real-time qPCR; Primers 5ʹ - GAGGAACAGTGGGGAAAATATCG -3ʹ (Forward) and 5ʹ - TGGGGTTACAGTGTCGGAAAG’ (Reverse) were used for amplification of the entire human PTH2R coding sequence (GenBank accession number NM_005048), sequences used for human GAPDH were GGAGCGAGATCCCTCCAAAAT -3ʹ (Forward) and 5ʹ- GGCTGTTGTCATACTTCTCATGG’ -3ʹ (Reverse). The 2^−ΔΔCt^ method was used to calculate gene expressions.

### Plasmid transfection

The PTH2R gene was amplified from HEK293T by standard PCR and then subcloned into pcDNA3.1-HA vector. All plasmids were sequenced. Lipofectamine 2000 (Invitrogen) was used for transfection, according to manufacturer's instructions.

### Cell proliferation detection

The CCK-8 assay (CCK-8 SAB Biotech. College Park, MD, USA) was used to detect cell proliferation. According to the manufacturer’s protocols, the cells were seeded into six-well plates at a density of 1.0 × 10^5^ cells per well, and were then cultured in medium supplemented with 5% FBS for 24 h (at 37 °C and 5% CO_2_). Then, 24 h after transfection, the cells were digested with trypsin and inoculated in triplicate into 96-well plates (3 × 10^4^ cells per well). Each well was incubated with 10 μL/well CCK-8 solution for 2 h every day, for a total of 5 d. The optical density at 450 nm was measured on a microplate reader. Three independent replications were performed.

### Transwell invasion and migration experiments

Transwell experiments were divided into transwell migration and transwell invasion experiments. The basic operations were as follows: transwell cells were placed into a 24-well culture plate, the chamber is referred to herein as the superior chamber and the culture plate is referred to as the lower compartment. The cells were then digested in a serum-free medium, following which the cell density was adjusted to 1 × 10^6^ cells/mL and the sample was inoculated in the upper chamber. Dulbecco's Modified Eagle Medium (DMEM) containing 10% FBS was then added to the lower chamber. Transwell invasion assays were performed by precoating the upper membrane with 40 µL of matrix glue (BD Biosciences, USA); the cells were fixed with 4% paraformaldehyde and were washed with phosphate buffer solution (PBS) after 24 h. Then, they were stained with 0.1% crystal violet (Solarbio, China) and representative images were observed at × 100 and × 200 magnification with an optical microscope (Olympus, Tokyo, Japan). Five non-repeating fields per chamber were selected for photography and counted.

### Clone formation experiment

The cells were digested with 0.25% trypsin until individual cells were obtained; the cell suspension was then diluted to a concentration of 1 × 10^4^ cells/mL. Then, 1,500 cells from the medium were added to each well of a six-well plate and incubated at 37 °C in 5% CO_2_. When the clones are visible to the naked eye in the six-well plate, cell culture was stopped and the cells were fixed in 4% paraformaldehyde for 15 min. Crystal violet staining was then performed, and the number of clones for which there were more than 50 cells was counted using an optical microscope.

### Immunohistochemical test

Immunohistochemistry (IHC) was conducted according to the antibody supplier's instructions. Sections of clinical samples were incubated overnight at 4 °C with a PTH2R primary antibody at different dilution ratios. Images were captured at appropriate magnification under an optical microscope (Nikon Microsystems, Shanghai, China). The antibody used in this study was anti-PTH2R (Chemicon International and GenWay Biotech).

### Western blot

Total cellular proteins were extracted using radioimmunoprecipitation assay (RIPA) lysates (Beyotime, Shanghai, China). Proteins were isolated and transferred to polyvinylidene fluoride (PVDF) membranes (Millipore, Temecula, CA, USA) by using 7.5% or 10% sodium dodecyl sulfate–polyacrylamide gel electrophoresis (SDS-PAGE). The membrane was sealed with primary resistance to PTH2R (Chemicon International and GenWay Biotech) at 4 °C overnight, following which the membrane was then washed and incubated with secondary antibodies. Protein bands were detected using enhanced chemiluminescence (Thermo Scientific Carlsbad, CA, USA).

### Statistical analysis

All data processing and analysis were completed by R software (version 4.0.2). For the comparison of the two groups of continuous variables, the statistical significance of the normally distributed variables was estimated using the independent Student t test, and the differences between the non-normally distributed variables were analyzed using the Mann–Whitney U test (i.e. Wilcoxon rank-sum test). The Chi-square test or Fisher's exact test was used to compare and analyze the statistical significance between the two groups of categorical variables. P < 0.05 was considered statistically significant.

## Results

### High expression of PTH2R gene in tumors

**Fig. 1:**
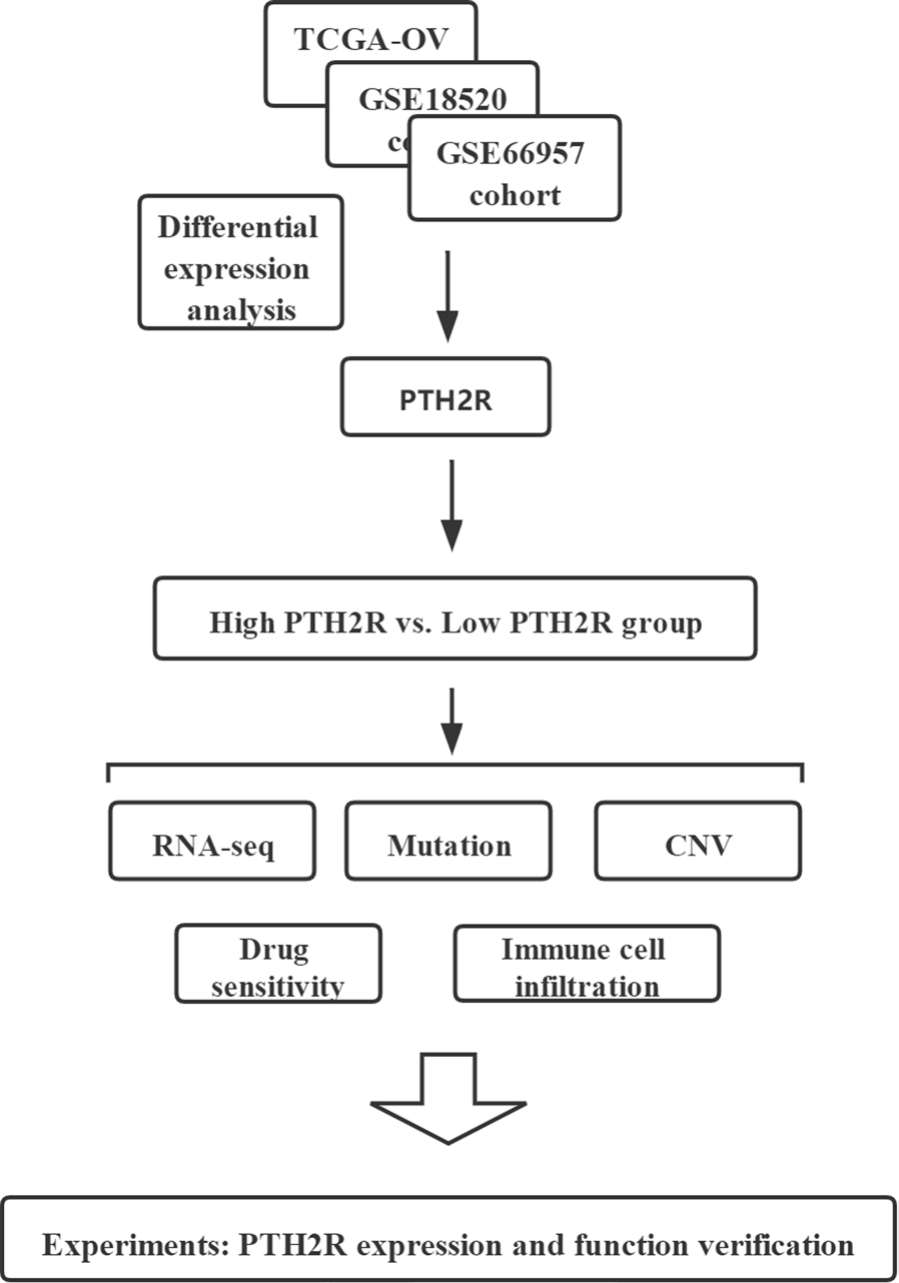
The visual flow-process diagram of this study

**Fig. 2:**
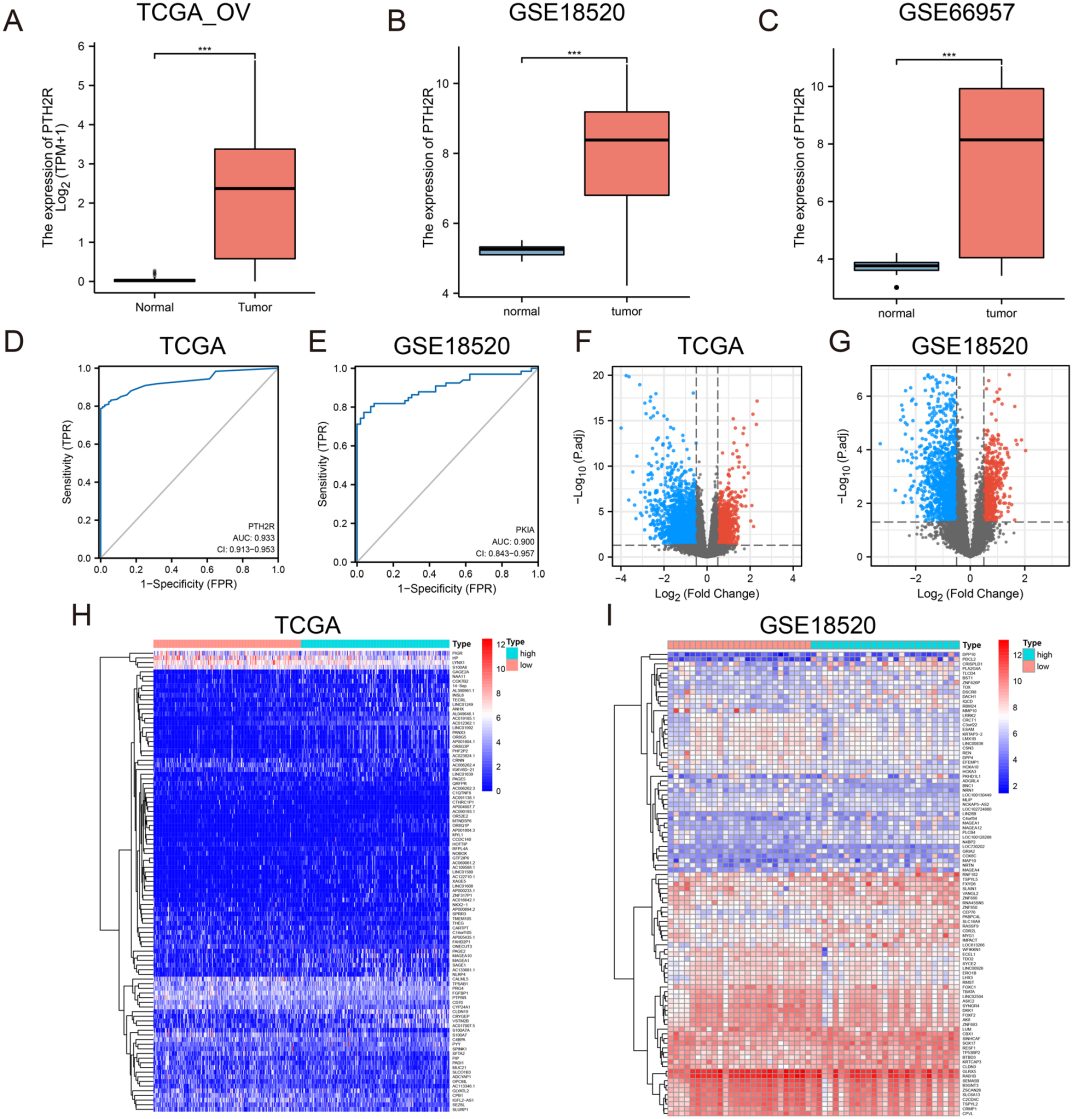
PTH2R is overexpressed in tumor. **A** The TCGA_OV dataset revealed that PTH2R was highly expressed in tumor tissues. **B** The GSE18520 dataset revealed that PTH2R was highly expressed in tumor tissues. **C** The GSE66957 dataset revealed that PTH2R was highly expressed in tumor tissues. **D–E** The ROC curve of TCGA_OV and GSE18520 dataset according to the expression of PTH2R. FI. The volcano plots and heatmaps showed the differentially expressed genes in TCGA_OV and GSE18520

The work flow is shown in Fig. 1. Screening and sorting of ovarian cancer data from the GEO database and sequencing data from TCGA (combined with the GTEx dataset) revealed that PTH2R was differentially expressed in the GSE18520, GSE66957 and TCGA-OV datasets; it was significantly highly expresses in tumor tissue (Fig. 2A–C). PTH2R, which is also known as PTHR2, has recently been found to regulate intracellular calcium and influence keratinocyte differentiation [39]. To date, PTH2R has not been studied in ovarian cancer, however, so here PTH2R was chosen as the study object. The function of PTH2R was explored and verified through subsequent analyses and experiments.

ROC analysis showed that the samples of the two datasets (GSE18520 and TGCA-GTEx) could be better distinguished after grouping them according to the expression of PTH2R (Fig. 1D, E). Comparing the DEGs in the high and low PTH2R expression groups revealed that the 2,448 genes were differentially expressed in GSE1850, while 1,984 DEGs found in TCGA-OV (Fig. 2F–I).

### Mutation and copy number variation analysis of PTH2R

**Fig. 3:**
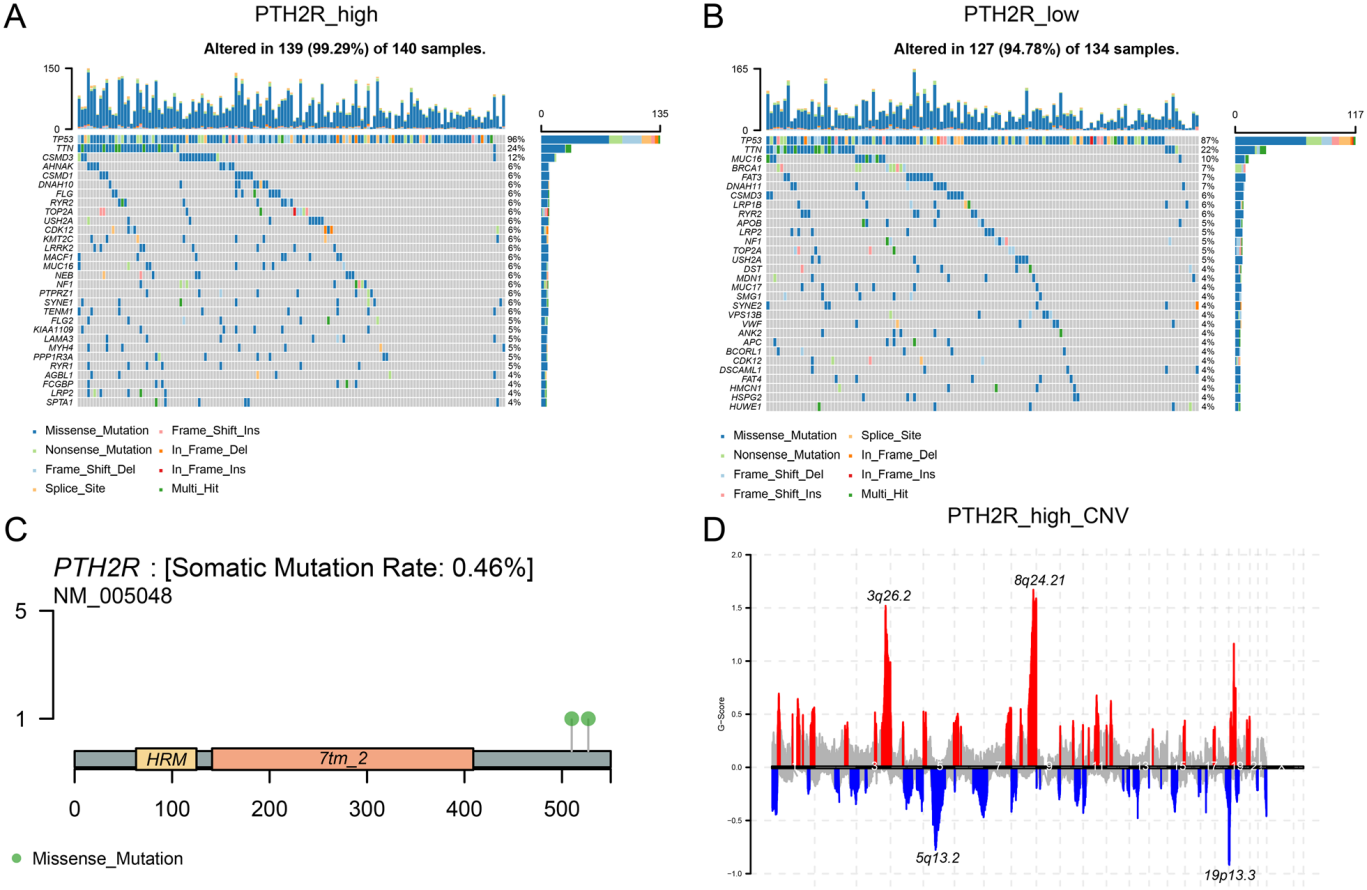
The alteration of mutations and copy number variation in high PTH2R expression group and low PTH2R expression group. **A** The top 30 mutation genes in high PTH2R expression group. **B** The top 30 mutation genes in low PTH2R expression group. **C**. The mutation sites information of PTH2R. **D** The CNV in the PTH2R high expression group

In the high PTH2R expression group, significant mutations were observed in the TP53, TTN, CSMD3, AHANK and DNAH10 genes; they were 96%, 24%, 12%, 6% and 6%, respectively. In the low PTH2R expression group, significant mutations were observed in the TP53, TTN, MUC16, BRCA1and FAT3 genes; they were 87%, 22%, 10%, 7% and 7% (Fig. 3A–B). Based on the mutation sites of PTH2R, the mutation information could be plotted (Fig. 3C).

In addition, by collating CNV information of TCGA-OV, it was possible to calculate CNV changes in the high PTH2R expression group (using GISTIC 2.0; Fig. 3D), revealing that the 3q26.2, 5q13.2, 8q24.21 and 19p13.3 locus of said group changed significantly.

### WGCNA

**Fig. 4:**
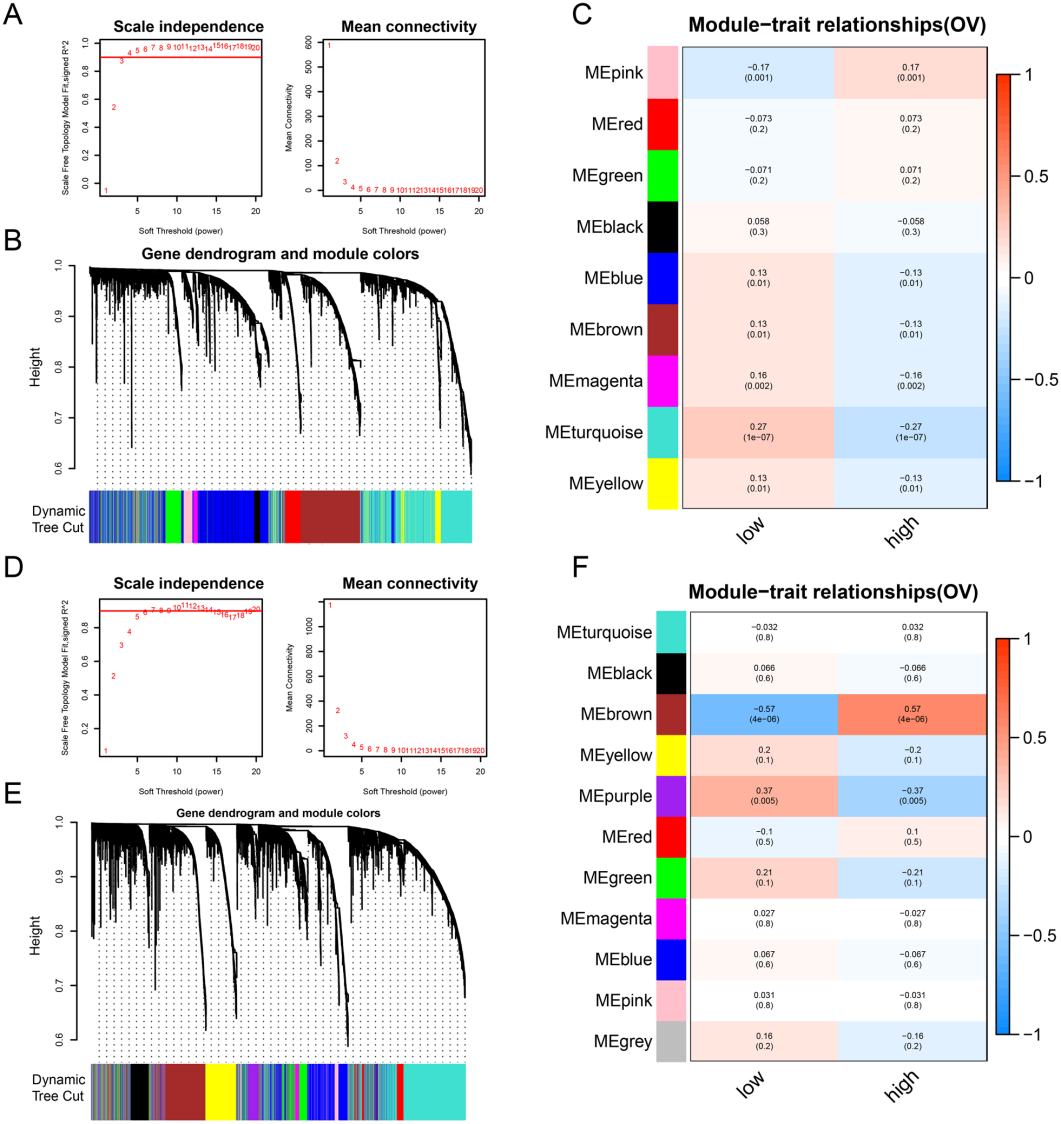
WGCNA analysis. **A** The lowest power for which scale independence in TCGA_OV dataset. **B** Repeated hierarchical clustering tree. **C** The associations between phenotypes and the modules in TCGA_OV dataset. **E** The lowest power for which scale independence in GSE18520 dataset. **F** Repeated hierarchical clustering tree in GSE18520. **G** The associations between phenotypes and the modules in GSE18520 dataset

By associating module feature genes with grouping information for both datasets, WGCNA revealed that 9 feature modules were determined in TCGA-OV. There were 3 modules with significant positive correlations and 6 modules with significant negative correlations (Fig. 4A, B). Furthermore, 11 feature modules were determined in GSE18520: there were 3 modules with significant positive correlations and 8 modules with significant negative correlations (Fig. 4D, E). The greater the correlation coefficient, the greater the correlation with PTH2R expression. Subsequently, the MEpink module with the largest correlation coefficient in TCGA-OV and the MEbrown module with the largest correlation coefficient in TCGA-OV were selected, as shown in Fig. 4C and F.

### Functional enrichment analysis

**Fig. 5:**
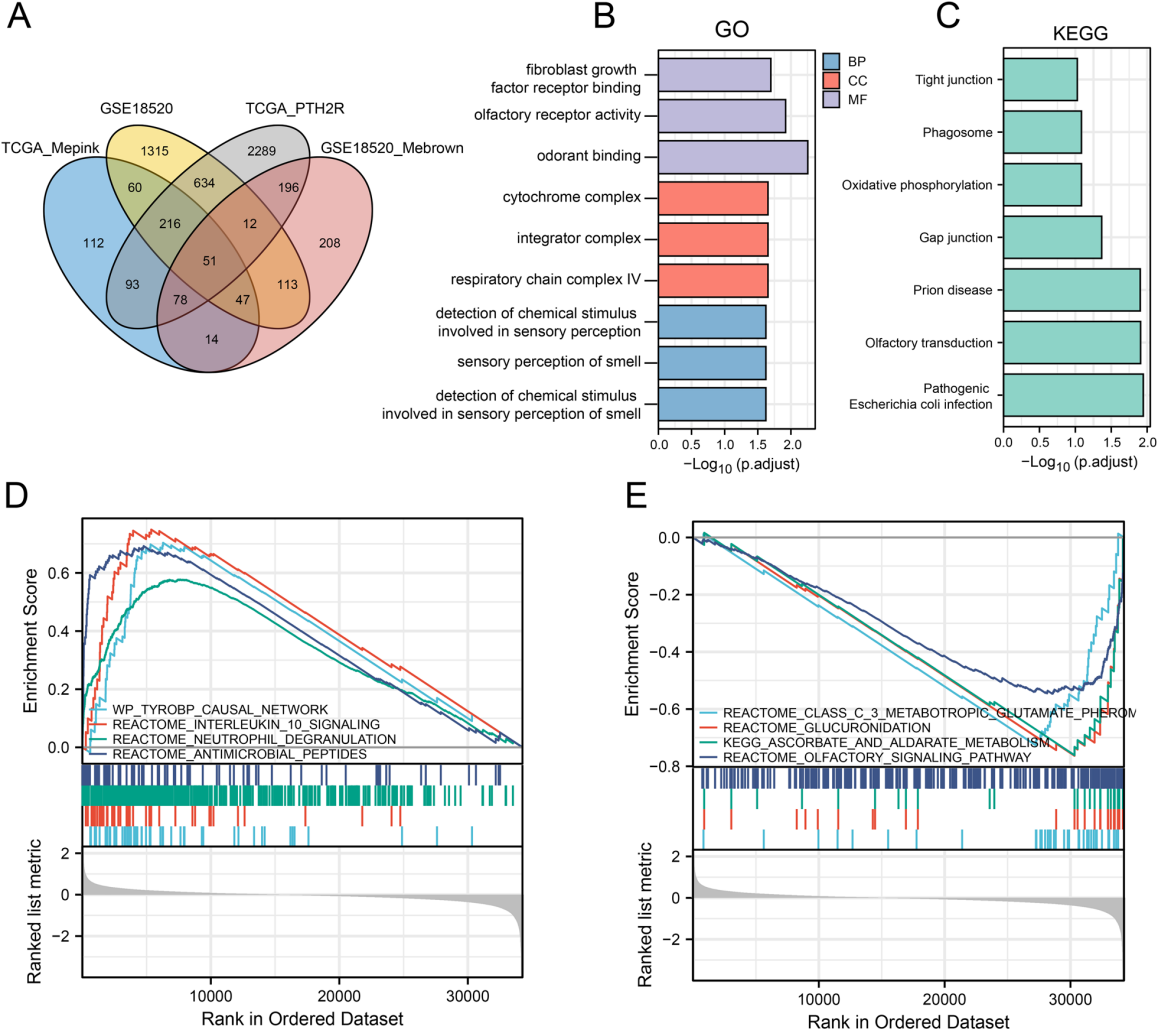
Enrichment analysis. **A** The Venn diagram of DEGs and WGCNA candidate genes. **B-C** GO and KEGG pathway enrichment of candidate 51 candidate genes. **D** the significant enriched pathway in high PTH2R expression with GSEA. **E** the significant enriched pathway in low PTH2R expression with GSEA

Intersecting the genes in the module with the previously obtained DEGs resulted in the identification of 51 hub genes. Functional enrichment analysis was conducted for these hub genes, with GO revealing that the DEGs were closely related to the detection of chemical stimulus involved in sensory perception of smell, sensory perception of smell, detection of chemical stimulus involved in sensory perception and snRNA 3'-end processing biological processes (Fig. 5B). KEGG functional analysis indicated that the differentially expressed genes mainly affected the Pathogenic Escherichia coli infection, Cardiac muscle contraction, Olfactory transduction and Amyotrophic lateral sclerosis pathways (Fig. 5C). The GSEA results showed that REACTOME_ANTIMICROBIAL_PEPTIDES, REACTOME_NEUTROPHIL_DEGRANULATION, REACTOME_INTERLEUKIN_10_SIGNALING etc. pathways were significantly enriched in high PTH2R group, while REACTOME_CLASS_C_3_METABOTROPIC_GLUTAMATE_PHEROMONE_RECEPTORS, REACTOME_GLUCURONIDATION etc. were mainly enriched in low PTH2R group (Fig. 5D, E).

### Drug sensitivity analysis and drug prediction

**Fig. 6:**
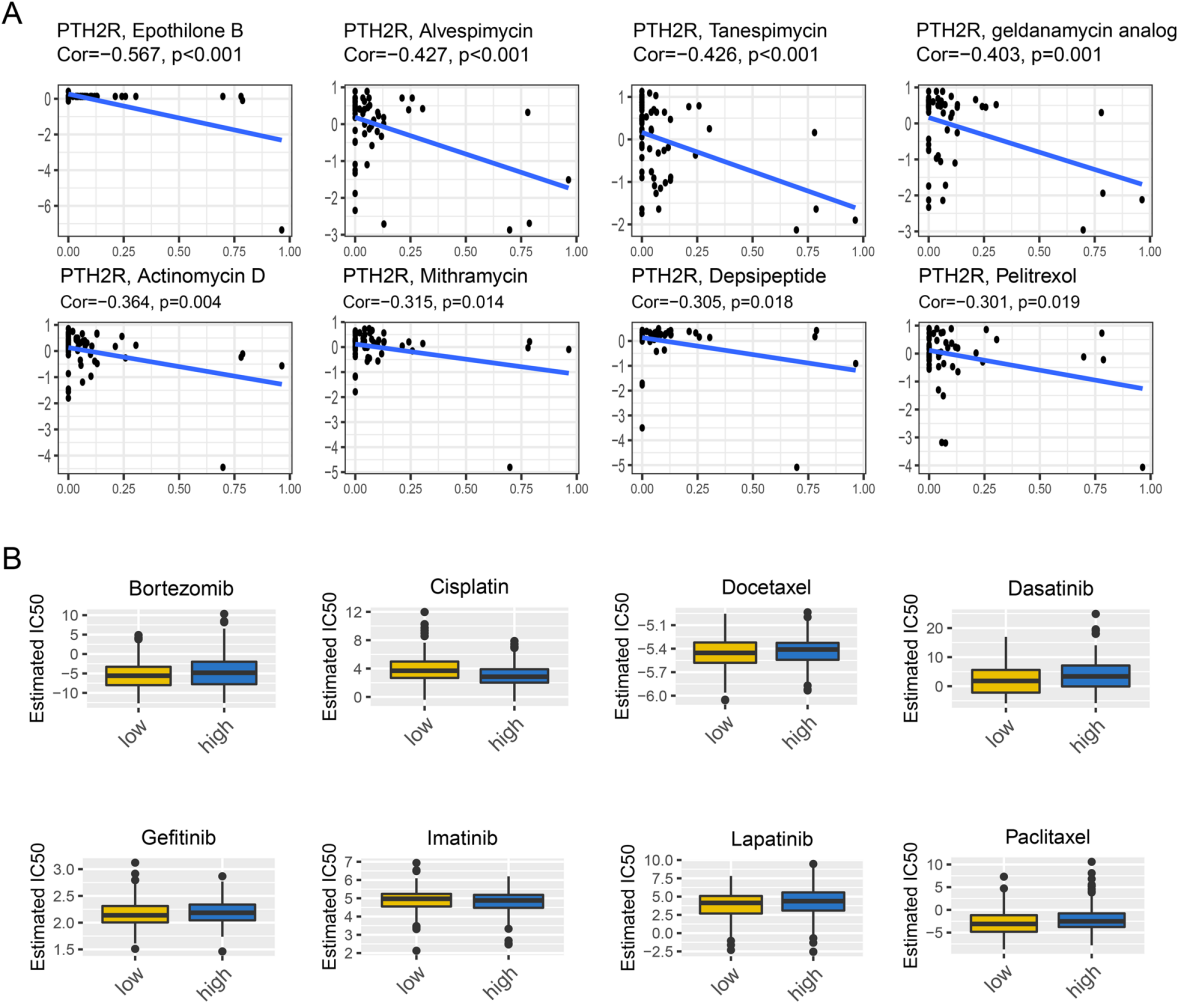
Drug sensitivity analysis. **A** The correlation between PTH2R expression and anti-tumor drugs based on the CellMiner database. **B** Drug prediction IC50 differences in PTH2R high and low group based on the GDSC database

Sorted by relevance, the top eight drugs most associated with PTH2R were selected. As shown in Fig. 6A, PTH2R was negatively correlated with Epothilone B, Alvespimycin, Tanespimycin, geldanamycin analog, Actinomycin D, Mithramycin, Depsipeptide and Pelitrexol.

In addition, IC50 value analysis of the high and low expression PTH2R groups using the GDSC database revealed that common anti-tumor drugs such as Docetaxel, Gefitinib had no significant difference, whereas Cisplatin, Lapatinib etc. did have a difference (Fig. 6B).

### Effect of PTH2R gene on immune cell infiltration in TCGA-OV patients

**Fig. 7:**
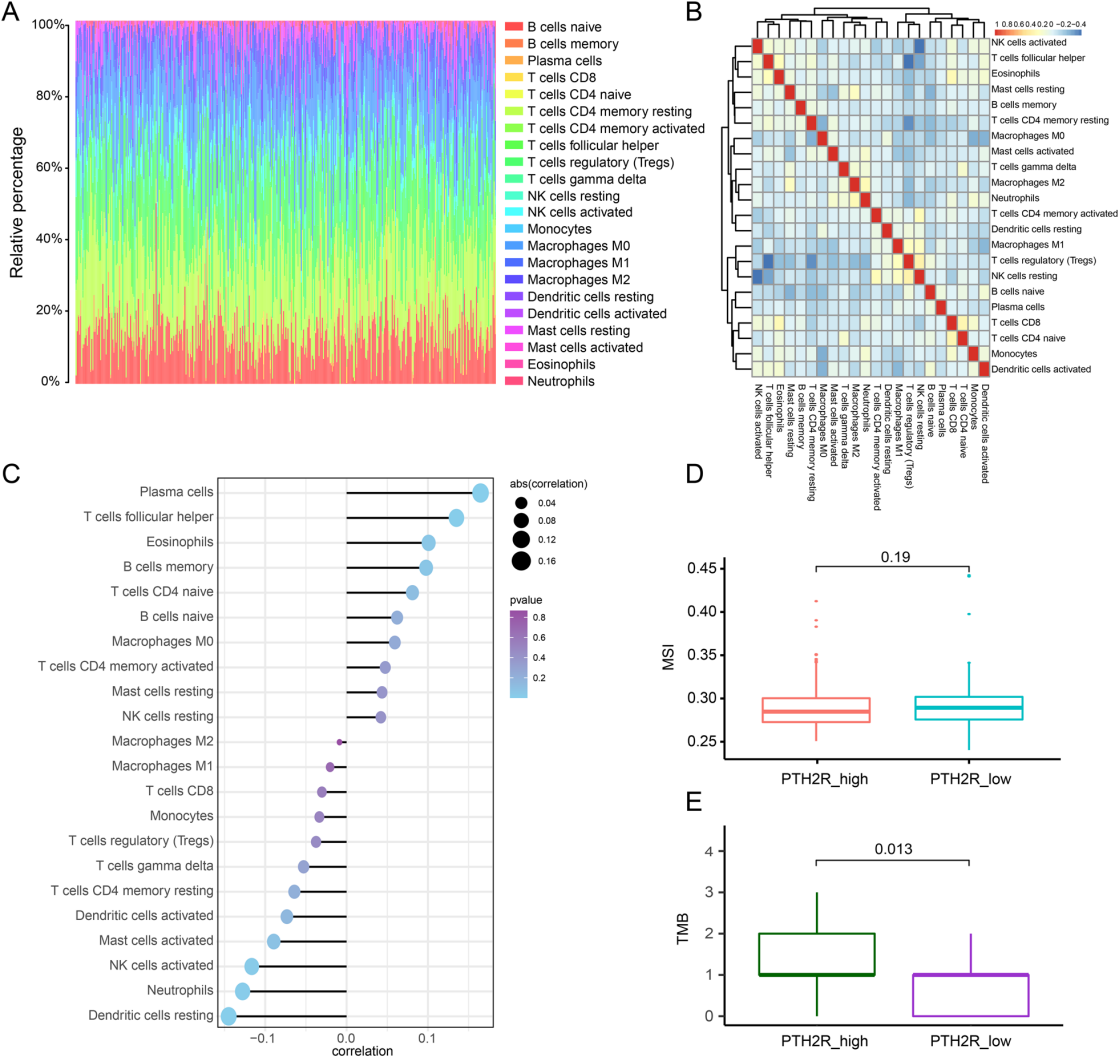
The landscape of immune microenvironment in TCGA_OV and PTH2R related immune cells correlation **A–B** The landscape of immune microenvironment in TCGA_OV and correlation between 22 immune cell infiltration score. **C** The correlation between PTH2R expression and infiltrated immune cells. **DE** The difference between MSI and TMB in PTH2R high expression and low expression group

To analyze the relationship between PTH2R gene expression and immune cell infiltration in TCGA-OV microenvironment, the proportion of immune cell invasion in the tumor microenvironment was calculated using the CIBERSORT algorithm. Figure 7A and B present landscape of immune cell infiltration in TCGA-OV tumor microenvironment and the correlation results of the immune cell score, respectively. PTH2R was found to be significantly positively correlated with Plasma cells, T cells follicular helper, Eosinophils etc. and negatively correlated with Dentritic cells resting, Neutrophils, NK cells activated.

Moreover, the comparison between high and low PTH2R expression group on TMB and MSI showed that TMB was significantly increased in the high PTH2R expression group ( p = 0.013), while the MSI showed no significant difference. (Fig. 7D and E).

### PTH2R gene expression verification

**Fig. 8:**
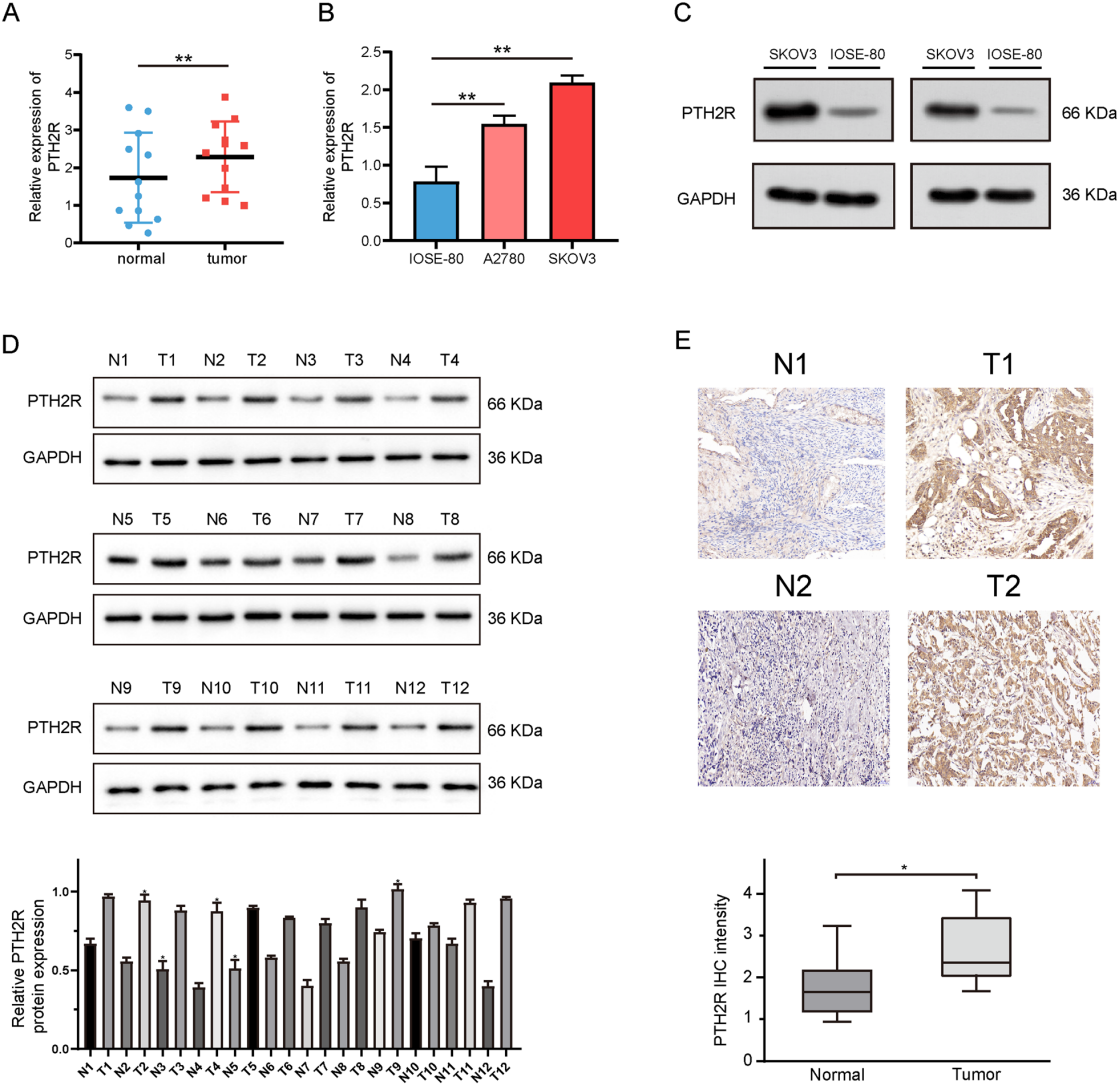
Validation of PTH2R expression in tissues and cells. **A–B** The qPCR showed that PTH2R is highly expressed in tumor tissues and cells. **C** The western blot showed that PTH2R protein is highly expressed in tumor cells. **D** The western blot showed that PTH2R protein is highly expressed in tumor tissues. **E** The IHC showed that PTH2R protein is highly expressed in tumor tissues. *p < 0.05, **p < 0.01

Comparing the PTH2R gene expression levels in ovarian cancer and normal tissues, and in the ovarian cancer cells and normal ovarian epithelial cells, revealed that the expression of PTH2R was significantly higher in ovarian cancer tissues and cells than in normal ovarian tissues and cells (Fig. 8A and B). Subsequently, western blot and IHC results also showed that PTH2R protein expression was significantly both higher in tumor than in normal tissues and cells (Fig. 8C–E, Additional file 1: Fig S1); this result is consistent with the expression trend of PTH2R in RNA-Seq data observed in the previous database.

### Inhibition of proliferation, invasion, and metastasis of ovarian cancer cells by PTH2R knockdown

**Fig. 9:**
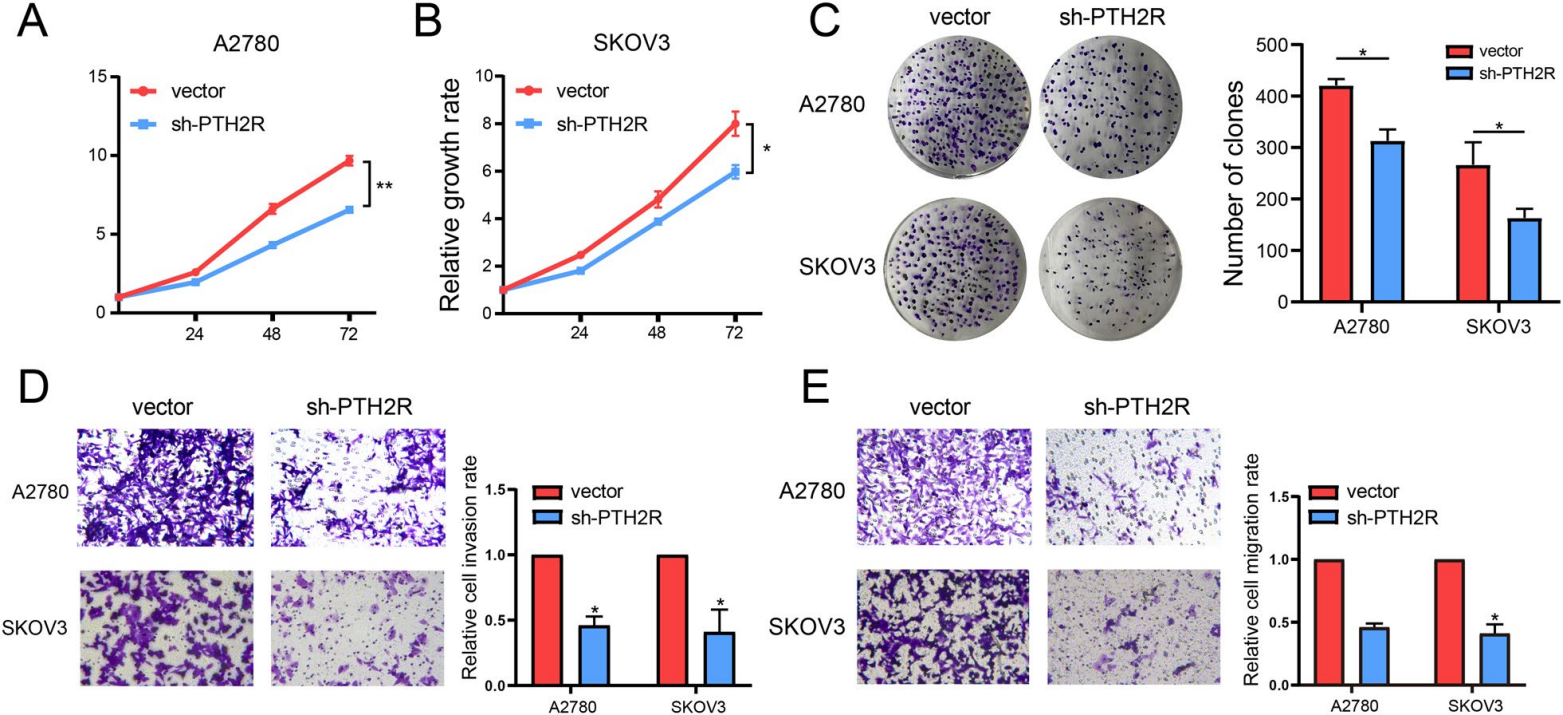
High expressed PTH2R is associated tumor proliferation, invasion and migration. **A**–**C** CCK-8 and clone formation assays showed that down regulation of PTH2R expression inhibited the proliferation of SKOV3 and A2780 cells. **D–E** Transwell assay showed that down regulation of PTH2R expression inhibited the invasion and migration of SKOV3 and A2780 cells

First, we tested the knockdown efficiency of PTH2R in ovarian cancer cells, and found that knocking down with sh-PTH2R reduced the expression of PTH2R more than half in A2780 and SKOV3 cell. (Additional file 2: Fig. S2A and B) The proliferation ability of PTH2R on ovarian cancer cells was detected by CCK-8 assay, revealing that compared with the control group, the decreased expression of PTH2R reduced the A2780 and SKOV3 proliferation activity (Fig. 9A and B). Similarly, the colony formation assay revealed that the downregulation of PTH2R inhibited the colony formation of A2780 and SKOV3 cells, compared with the control group (Fig. 9C). Subsequently, transwell invasion and migration experiments further demonstrated that PTH2R downregulation significantly reduced the invasion and migration of tumor cells (Fig. 9D and E). In conclusion, the PTH2R gene was found to be involved in the proliferation, invasion, and metastasis of ovarian cancer. This result further validates the results of the bioinformatical analysis.

## Discussion

Ovarian cancer is the most malignant cancer among diseases of the female reproductive system, accounting for more than 90% of ovarian cancer deaths. If epithelial ovarian cancer is identified at stage II or III, the estimated 5-year mortality rate is ~ 70% [40]. Currently, the standard treatment for patients with advanced ovarian cancer comprises cytolytic surgery and postoperative chemotherapy. However, the efficacy and prognosis of ovarian cancer patients remain poor due to poor tolerance to chemotherapy and the lack of effective monitoring measures. Thus, it is expected that new targets can be found for the diagnosis and treatment of ovarian cancer.

With the current progress in bioinformatics and the development of sequencing technology, an increasing number of researchers are gaining access to larger sample sets from public databases from which to mine valid information. In this study, the combined TCGA-OV data set and the GSE18520, GSE66957 data sets showed that PTH2R was significantly elevated in ovarian cancer tissues. PTH2R, also known as PTHR2 (Ensembl ID: ENSG00000144407), was first reported as a selective parathyroid hormone receptor [41]. Although PTH2R was identified as prognostic index in papillary thyroid cancer and breast cancer bone metastases [42, 43]. there are no PTH2R-related studies in ovarian cancer. Moreover, we surveyed the expression of PTH2R in pan-cancer via the GEPIA 2 database (http://gepia2.cancer-pku.cn), and noticed that PTH2R is differentially expressed in several cancers, like glioblastoma, low-grade glioma, kidney chromophobe and ovarian cancer. However, PTH2R tends to be overexpressed only in ovarian cancer.

Then, tumor samples were further divided into high and low PTH2R expression groups, which allowed PTH2R-related genes and molecular characteristics to be better investigated. Analyzing the mutation characteristics of the high and low PTH2R expression groups revealed that there were significant mutation differences between the two. In the high PTH2R expression group, the CSMD3, AHANK, CSMD1 etc. mutations were more frequent, while in the low expression group, the MUC16, BRCA1, FAT1 etc. mutations were more frequent. In addition, CNV changes were also identified in high PTH2R expression group, and significant changes were identified at 3q26.2, 5q13.2, 8q24.21 and 19p13.3 locus. Analysis of drug resistance revealed that resistance to Cisplatin and Imatinib was more obvious in the high PTH2R expression group.

Moreover, with the development of immunotherapy, here the relationship between PTH2R and immune cell infiltration was investigated, alongside the prospect of immunotherapy, by combining RNA-Seq data and a widely used immune cell scoring algorithm. Comparing the correlations between the expression of PTH2R and immune cell infiltration revealed that PTH2R showed a significant positive correlation with Plasma cells, but was negatively correlated with Dendritic cells resting. However, the coefficient of association was relatively low, so this does not necessarily mean that PTH2R is definitely related to immune response; it also may be due to insufficient of the sample size. In addition, TMB and MSI scores showed that the TMB was significantly increased in the high PTH2R expression group, which may be a potential diagnostic marker.

Although bioinformatics analysis provided important information in this PTH2R study, experiments were still needed to verify the expression and function of PTH2R. Therefore, the mRNA expression and protein levels of PTH2R were investigated through qPCR, WB, and IHC, revealing that it was consistent with the expression trend identified in the databases. In other words, the expression of PTH2R in tumors was significantly higher than that in normal tissues and cells. In addition, a series of tumor cell function experiments, such as CCK-8, clonogenesis, and transwell assays, showed that PTH2R knockdown significantly inhibited the growth, invasion, and migration of tumor cells. In conclusion, PTH2R is expected to become a new molecular marker for ovarian cancer.

This study is the first to report the expression and function of PTH2R in ovarian cancer. Combined with molecular information available in public databases, this study deeply explored the function and mechanism of PTH2R from the perspective of multiple omics; its findings provide an important preliminary basis for future PTH2R-related research. However, there are some limitations in this study, as only the expression and biological function of PTH2R were verified; it was not possible to completely characterize the influences of drug resistance and mutation characteristics. The future research should focus on elucidating the mechanism to understand how PTH2R influenced cell proliferation and migration.

## Conclusion

In this study, PTH2R was found to be highly expressed in ovarian cancer through bioinformatics methods, combined with the GEO and TCGA databases. Experiments verified that PTH2R was not only highly expressed in tumor cells and tissues, but also affected the proliferation, invasion, and migration of tumor cells. PTH2R is thus expected to become a new molecular marker for ovarian cancer.

## Supplementary Information

**Additional file 1:** **Figure S1.** IHC plots of PTH2R protein expression in remaining paired tissues.

**Additional file 2:** **Figure S2.** Knockdown efficiency of PTH2R in ovarian cancer cells. A. Knockdown efficiency of PTH2R in A2780 cells. B. Knockdown efficiency of PTH2R in SKOV3 cells.

## Data Availability

The datasets used and/or analyzed during this study can be acquired from the corresponding author upon reasonable request.

## Abbreviations

BP: Biological process
CCK-8: Cell counting kit 8
CC: Cellular component
CNV: Copy number variation
DEGs: Differentially expressed genes
DMEM: Dulbecco's modified eagle medium
FDR: False discovery rate
GEO: Gene expression omnibus
GO: Gene ontology
GSEA: Gene set enrichment analysis
GDC: Genomic data commons
GDSC: Genomics of drug sensitivity in cancer
IHC: Immunohistochemistry
KEGG: Kyoto encyclopedia of genes and genomes
mRNA: Messenger ribonucleic acid
MSI: Microsatellite instability
MF: Molecular function
PTH2R: Parathyroid hormone 2 receptor
PBS: Phosphate buffer solution
PVDF: Polyvinylidene fluoride
qPCR: Quantitative polymerase chain reaction
RIPA: Radioimmunoprecipitation assay
ROC: Receiver operating characteristic
RNA-Seq: RNA sequencing
TCGA: The cancer genome atlas
TOM: Topological overlap metric
TMB: Tumor mutation burden
WGCNA: Weighted gene co-expression network analysis
WB: Western blot
