# Supplementary figures and images for: PTH2R is related to cell proliferation and migration in ovarian cancer: a multi-omics analysis of bioinformatics and experiments
Source: Cancer Cell Int. 2022 Apr 11;22:148. doi: 10.1186/s12935-022-02566-2 (PMC8996580; doi:10.1186/s12935-022-02566-2)

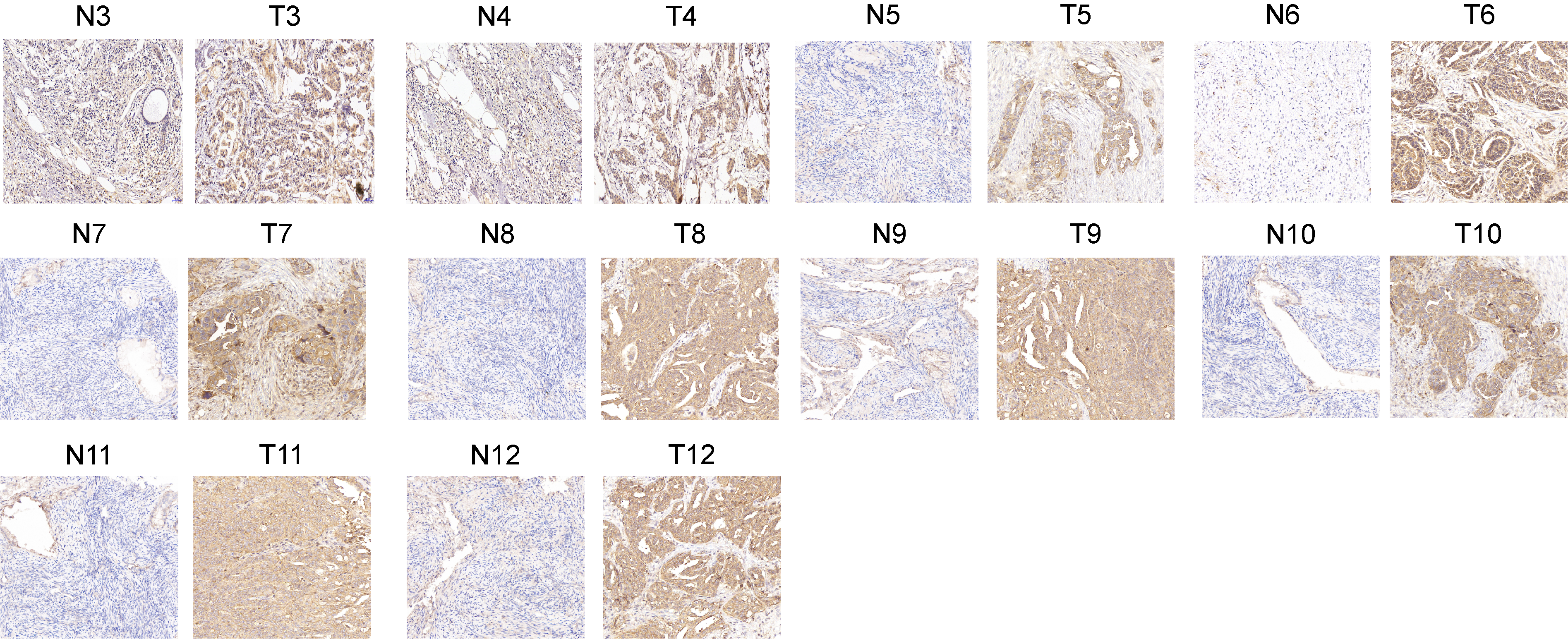

Supplement: Supplementary file 1 — Additional file 1: Figure S1. IHC plots of PTH2R protein expression in remaining paired tissues. [file 12935_2022_2566_MOESM1_ESM.tif]

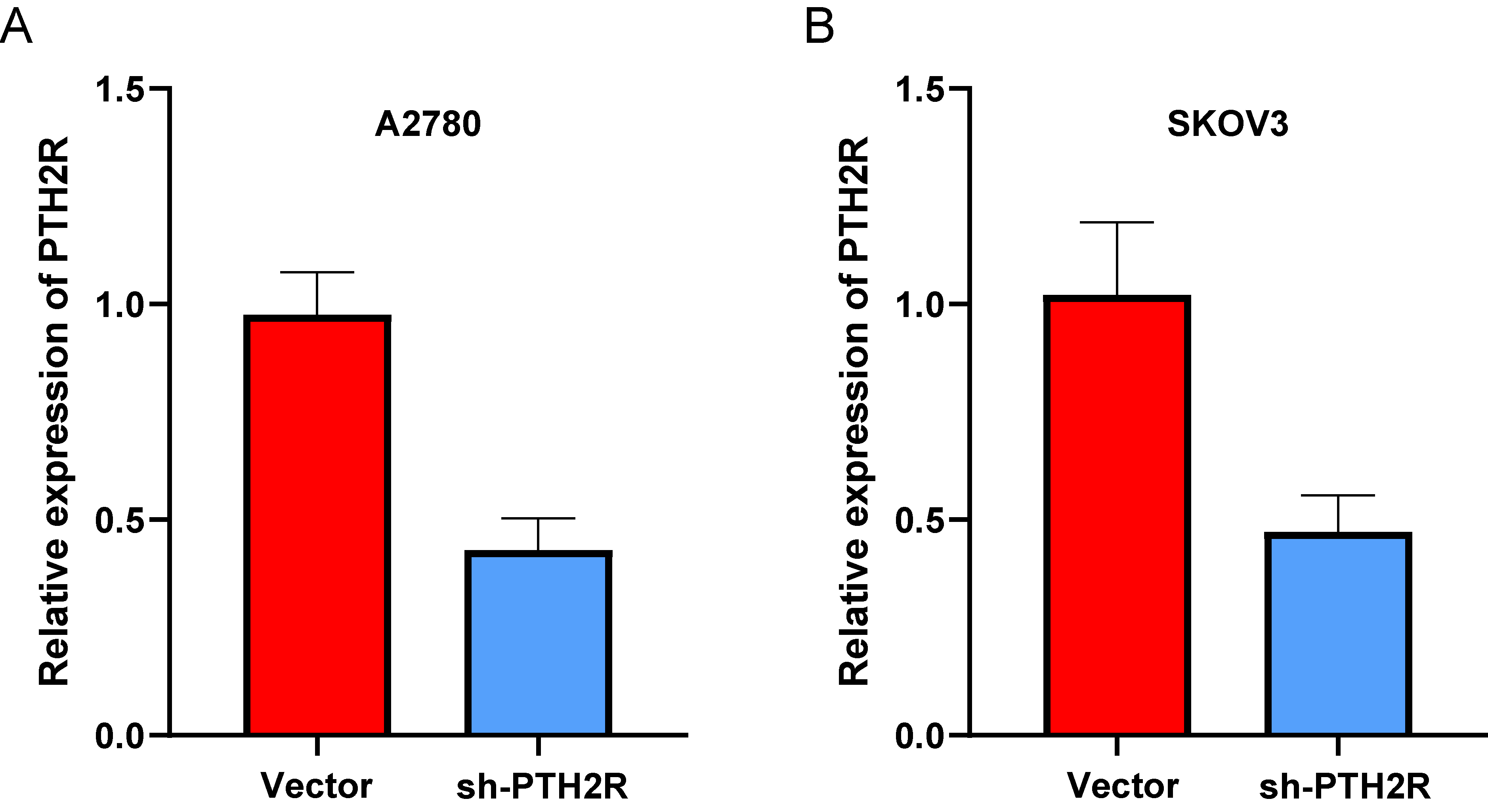

Supplement: Supplementary file 2 — Additional file 2: Figure S2. Knockdown efficiency of PTH2R in ovarian cancer cells. A. Knockdown efficiency of PTH2R in A2780 cells. B. Knockdown efficiency of PTH2R in SKOV3 cells. [file 12935_2022_2566_MOESM2_ESM.tif]
